# H19 Sperm Methylation in Male Infertility: A Systematic Review and Meta-Analysis

**DOI:** 10.3390/ijms24087224

**Published:** 2023-04-13

**Authors:** Rossella Cannarella, Andrea Crafa, Federica Barbagallo, Scott D. Lundy, Sandro La Vignera, Rosita A. Condorelli, Aldo E. Calogero

**Affiliations:** 1 Department of Clinical and Experimental Medicine, University of Catania, 95123 Catania, Italy; 2 Glickman Urological & Kidney Institute, Cleveland Clinic Foundation, Cleveland, OH 44125, USA

**Keywords:** H19, methylation, oligozoospermia, infertility, epigenetic

## Abstract

This systematic review and meta-analysis summarize the difference in the methylation of the *H19* gene in patients with abnormal versus normal conventional sperm parameters. It also evaluates the effects of age and sperm concentration on *H19* methylation in spermatozoa using meta-regression analysis. It was performed according to the MOOSE guidelines for meta-analyses and Systematic Reviews of Observational Studies and the Preferred Reporting Items for Systematic Review and Meta-Analysis Protocols (PRISMA-P). The quality of the evidence reported in the studies included was assessed using the Cambridge Quality Checklists. A total of 11 articles met our inclusion criteria. Quantitative analysis showed that *H19* methylation levels were significantly lower in the group of infertile patients than in fertile controls. The reduction in methylation was much more pronounced in patients with oligozoospermia (alone or associated with other sperm parameter abnormalities) and in those with recurrent pregnancy loss. Meta-regression analysis showed the results to be independent of both patient age and sperm concentration. Therefore, the *H19* methylation pattern should be evaluated among couples accessing assisted reproductive techniques (ART), in order to gain prognostic information on ART outcome and offspring health.

## 1. Introduction

According to the World Health Organization (WHO), infertility is a complex disease that is caused by numerous factors impairing either the male or female, or both partners. Defined by the failure to achieve pregnancy after 12 or more months of regular unprotected intercourse [1], more than 48.5 million couples suffer from infertility globally [2]. The cause of infertility recognizes a male factor alone or in combination with a female factor in at least half of all cases. It is estimated that there are more than 30 million infertile men worldwide [2]. Despite the alarming prevalence, the causes of male infertility remains enigmatic in a large percentage of cases. In fact, a prospective study of 1737 infertile patients with abnormal seminal parameters identified the etiology of infertility in only 40% of men [3]. This lack of definitive diagnosis is particularly common for oligozoospermic men, with approximately 75% of oligozoospermic patients diagnosed as idiopathic [3]. This evidence motivates the urgent need for further research to explain the apparently idiopathic cases.

In recent years, increasing attention has been paid to the role of epigenetic modifications in the pathogenesis of human disease and male infertility in particular. Epigenetics represents the set of reversible gene modifications that influence expression and regulation without altering the underlying DNA sequence [4]. These modifications include cytosine methylation, histone tail modifications, and short and long-noncoding RNAs (ncRNAs) [4], among others. We know that the correct regulation of these epigenetic mechanisms during gonadal development and spermatogenesis plays a fundamental role in normal sperm function, production, and male fertilization potential. Consequently, several factors, such as protamine abnormalities, the presence of endocrine-disrupting chemicals, or diet, have been implicated in determining epigenetic modifications [4,5]. Since numerous epigenetic modifications occur during spermatogenesis to produce the highly specialized haploid cells necessary for reproduction, spermatozoa are highly susceptible to epigenetic modifications, and such modifications could potentially explain a large proportion of idiopathic infertility cases [6].

DNA methylation is among the most studied epigenetic changes in male infertility. Indeed, this process is essential to ensure proper chromatin condensation in the sperm head, enabling sperm maturation and its capacity to be involved in fertilization and post-fertilization events. Of particular importance is the methylation process of differentially-methylated regions (DMRs) of imprinted genes. Imprinted genes are those in which one allele (either maternal or paternal) is expressed while the other is repressed. This process regulates the maternal or paternal expression of a specific gene, allowing for the expression of only one of the two in the offspring [6]. Changes in the normal methylation pattern of these genes have been associated with impaired fertility and the risk of transmitting epigenetic abnormalities to the offspring [7].

The *H19*/*insulin-like growth factor 2* (*IGF2*) genes are among the most studied imprinted genes. They share enhancers and DMRs located downstream and upstream of the *H19* gene, respectively. Normally, DMRs of the *H19* gene are methylated in spermatozoa and are unmethylated in oocytes, and somatic cells express the maternal *H19* and the paternal *IGF2* alleles [8]. When confirming the role of *H19*/*IGF2* methylation aberrations in the pathogenesis of male infertility, meta-analytic data showed lower DMR methylation levels in the *H19* gene in infertile male patients and a 9.91-fold higher risk of DMR aberration in the *H19* gene in these patients compared to fertile controls [9]. Furthermore, aberrations in the methylation of the *H19*/*IGF2* genes have been associated with higher rates of sperm DNA fragmentation (SDF). Finally, it has been observed that lower levels of *H19* gene methylation are associated with higher rates of recurrent miscarriages [10].

Recently, increasing attention has been paid to the role of age in the decline in sperm function and male fertility. A recent meta-analysis showed an age-related decline in semen volume, total sperm motility, progressive motility, and normal morphology [11]. Additionally, older age appears to be associated with a higher SDF rate than younger age [12]. Furthermore, the literature has shown a close association between aging and epigenetic changes [13]. Limited and conflicting evidence is available on the role of age regarding the different methylation patterns of the *H19*/*IGF2* genes [14,15]. So far, only a meta-analysis published in 2017 has addressed this issue [9]. However, a comprehensive meta-regression analysis to explore whether the methylation status of these genes changes with age or sperm concentration has never been performed. Thus, this meta-analysis aims to provide an update on the difference in the *H19* gene DMR methylation of patients with abnormal versus normal conventional sperm parameters, and to evaluate the effects of age and sperm concentration on the *H19* gene methylation rate in human spermatozoa.

## 2. Materials and Methods

### 2.1. Search Strategy

The meta-analysis was performed according to the MOOSE guidelines for Meta-analyses and Systematic Reviews of Observational Studies [16] and the Preferred Reporting Items for Systematic Review and Meta-Analysis Protocols (PRISMA-P) [17]. The MOOSE and PRISMA checklists have been included in Supplementary Tables S1 and S2. Articles were searched for on Pubmed and Scopus databases from the year of their founding until November 2022. The search strategy used the following combination of MeSH terms and keywords: “H19”, “CTCF6”, “CTCF3”, “IGF2”, “gene methylation”, “fertilization rate”, “sperm DNA fragmentation”, “assisted reproductive technique”, “pregnancy rate”, “abortion” and “miscarriage”. Additional manual searches were conducted using the reference lists of relevant studies. Only human studies and original articles were selected. No language restriction was applied, since the abstracts of articles written in a language other than English and Italian were also available in English. The meta-analysis was registered on PROSPERO with the code CRD42023397056.

### 2.2. Selection Criteria

Articles were assessed for eligibility using the PECOS (Population, Exposure, Comparison/Comparator, Outcome, Study type) model system [18] (Table 1). The selection of eligible studies was carried out by two researchers (AC and RC). For each article, the eligibility assessment was performed by two reviewers (SLV and RAC) independently and unblinded. The titles and abstracts of the studies were first independently screened for inclusion. If there was any uncertainty, each researcher reviewed the full text to establish whether or not to include it. Any disagreement between the reviewers was resolved via discussion between the two reviewers. However, if no consensus was reached, another reviewer made the final decision (AEC). The selected articles finally underwent data extraction.

### 2.3. Data Extraction

The following information was extracted from the eligible studies: first author, year of publication, study design, the sample size of the cases and controls, the age of cases and controls, sperm concentration of cases and controls, *H19* methylation levels, and the methylation assessment method. In case of missing information in the original articles, the data of interest were extracted from the meta-analysis by Santi and colleagues [9], who performed a preliminary analysis. Since we found some discrepancies between the data reported by the authors of the eligible articles and those included in the meta-analysis by Santi and colleagues [9], we also decided to contact the manuscript authors directly to evaluate the accuracy of the data. If there was no response, we sent a reminder ten days after the first email and waited another two weeks before considering the data to be missing. Only data and information received by the authors via email were included in the final analysis reported in the present study.

### 2.4. Quality Assessment

The quality of evidence (QoE) of the studies was assessed by two researchers (AC and RC). Since all studies were cross-sectional, the QoE was assessed using the Cambridge Quality Checklists [19]. In detail, these are three checklists designed to identify high-quality studies of correlates, risk factors, and causal risk factors. The checklist for correlates evaluates the appropriateness of the sample size and the quality of the outcome measurements. The checklist for risk factors assigns high-quality scores only to those studies with appropriate time-ordered data. Finally, the checklist for causal risk factors evaluates the type of study design. To draw confident conclusions about correlates, the correlate score must be high. To draw confident conclusions about risk factors, both the checklists for correlates and risk factor scores must be high. To draw confident conclusions about causal risk factors, all three checklist scores must be high. Any disagreement between the two investigators was resolved through discussion with the other two researchers (SLV and RAC).

### 2.5. Statistical Analysis

Quantitative data analysis was performed using the Comprehensive Meta-Analysis Software (Version 3) (Biostat Inc., Englewood, NJ, USA). The standardized mean difference (SMD) was calculated for statistical comparison between cases and controls because the method of assessing methylation was different between studies. Statistical significance was accepted for *p*-values less than 0.05. The Cochran-Q and heterogeneity index (I^2^) were used to assess statistical heterogeneity. In particular, if I^2^ was less or equal to 50%, the variation in the studies was considered homogenous and the fixed effect model was adopted to calculate the pooled effect size. Conversely, if I^2^ was greater than 50%, there was significant heterogeneity between the studies, and the random effects model was used. Publication bias was qualitatively analyzed using the funnel plot skewness, which suggested that there were some missing studies on one side of the graph. For the quantitative analysis of publication bias, we used Egger’s intercept test, which assessed the statistical significance of the publication bias. In case of publication bias, unbiased estimates were calculated using the “trim and fill” method. Furthermore, a meta-regression analysis was performed to test the effect of different parameters on *H19* DMR SMD. Potential predictors were included as continuous variables, such as age and sperm concentration.

## 3. Results

**Figure 1:**
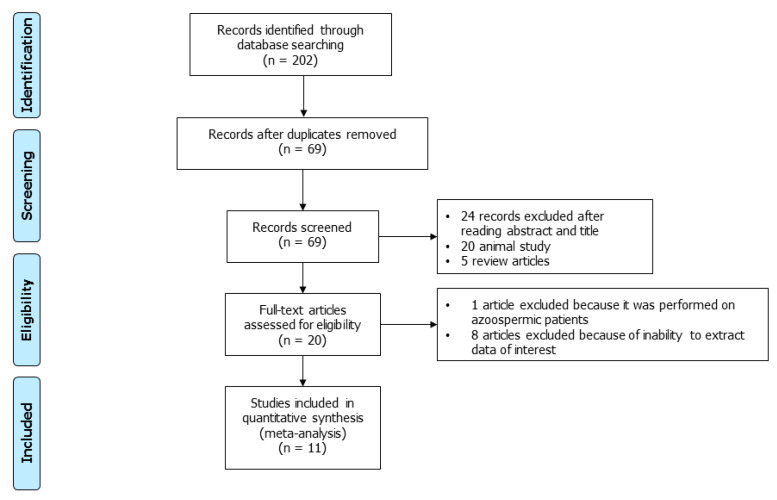
PRISMA flowchart of the included studies.

Using the above-mentioned search strategy, 202 articles were retrieved. After the exclusion of 133 duplicate records, 69 articles were screened. Of these, 24 were judged not pertinent after reading their abstracts or full texts. In addition, 20 animal studies and 5 review articles were excluded. The remaining 20 studies were carefully read and considered for inclusion in the analysis. Of these, 1 was excluded because it included azoospermic patients [20], 5 were included because the data of interest were present in the full text of the article [21,22,23,24,25], while, for the other 14, the corresponding author of the original article was contacted for information on missing data [26,27,28,29,30,31,32,33,34,35,36,37,38]. Of these, 9 replied to the email sent [26,27,28,29,30,31,32,33]. Finally, we were able to receive information on *H19* gene methylation from 6 articles [26,27,28,29,30,34]. Therefore, a total of 11 articles were included [21,22,23,24,25,26,27,28,29,30,34] (Figure 1). The main characteristics of the included studies are summarized in Table 2.

### 3.1. Quality of Evidence of Included Studies

All 11 included studies assessed with the Cambridge quality checklist scored <6 out of a total of 15. Although this scale does not establish a precise threshold for differentiating between high- or low-quality studies, the results suggest that the studies included are of relatively low methodological quality (Table 3).

### 3.2. Methylation Levels of H19 and Meta-Regression Analysis

All 11 included studies assessed the *H19* methylation levels [21,22,23,24,25,26,27,28,29,30,34]. In the analysis, the study by Dong and colleagues was considered 3 times, as patients with normozoospermia were compared with a group of patients with oligozoospermia, asthenozoospermia, and teratozoospermia [21]. The study by Li and colleagues was considered four times, as normozoospermic patients were compared with patients with oligozoospermia or asthenozoospermia in the original article. Moreover, in both comparisons, both the methylation of the CTCF-6 region and that of the whole gene were evaluated [22]. Similarly, the study by Peng and colleagues was considered twice, as patients with normozoospermia were compared with a group of patients with asthenoteratozoospermia or one oligozoospermia [24]. Finally, for the same reason, the study by Boisonnas and colleagues was considered twice, since normozoospermic patients were compared with a group of patients with teratozoospermia or with oligoasthenoteratozoospermia [34]. Due to the presence of significant heterogeneity, as demonstrated by the Q-test (Q-value = 163.459; *p*-value = 0.000) and I^2^ = 89.6%, the random effect model was used.

**Figure 2:**
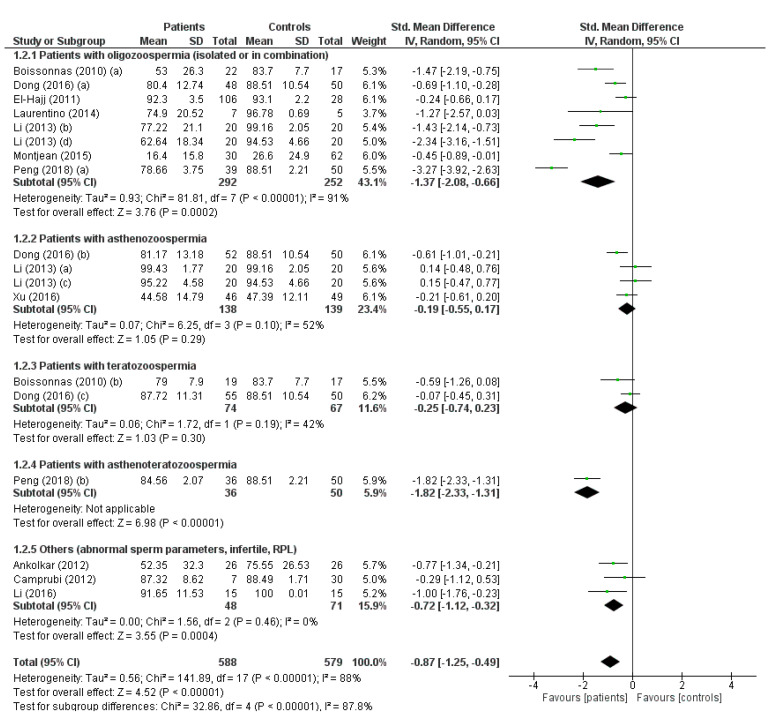
*H19* gene methylation in spermatozoa of patients with infertility/abnormal sperm parameters and controls. The studies meta-analyzed are: [21,22,23,24,25,26,27,28,29,30,34].

**Figure 3:**
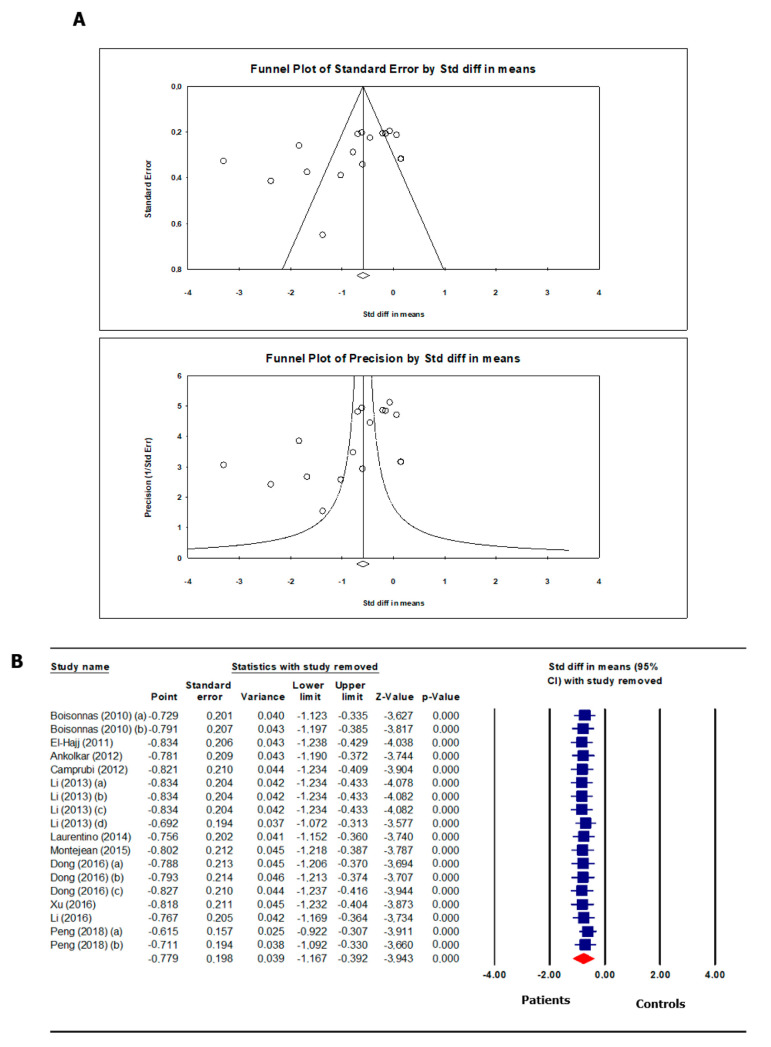
Funnel plot (**A**) and sensitivity analysis (**B**) of H19 gene methylation in spermatozoa of patients with infertility/abnormal sperm parameters and controls. The studies included in the funnel plot and in the sensitivity analysis are: [21,22,23,24,25,26,27,28,29,30,34]. Blue squares represent the standard difference of the mean after removal of the studies corresponding to the specific square. The read diamond is the overall standard difference of the mean without any study removal.

Overall, the analysis showed that *H19* methylation levels were significantly lower in the group of infertile patients than in the fertile controls (SMD −0.87, 95% CI −1.25, −0.49; *p* < 0.00001). Subgroup analysis showed that the reduction in methylation was much more pronounced in patients with oligozoospermia (alone or associated with other sperm parameter abnormalities) (SMD −1.37, 95% CI −2.08, −0.66; *p* = 0.0002) compared to patients with alterations other than oligozoospermia in their sperm parameters. Additionally, the methylation levels in patients with a history of infertility or recurrent pregnancy loss (RPL) were significantly reduced (SMD −0.87, 95% CI −1.12, −0.32; *p* = 0.0004) (Figure 2). There was evidence of publication bias, as shown by Egger’s test (intercept −4.90408, 95% CI −10.11456, 0.30640, *p* = 0.032) and funnel plot asymmetry (Figure 3A). No study was sensitive enough to alter the above-reported results (Figure 3B).

**Figure 4:**
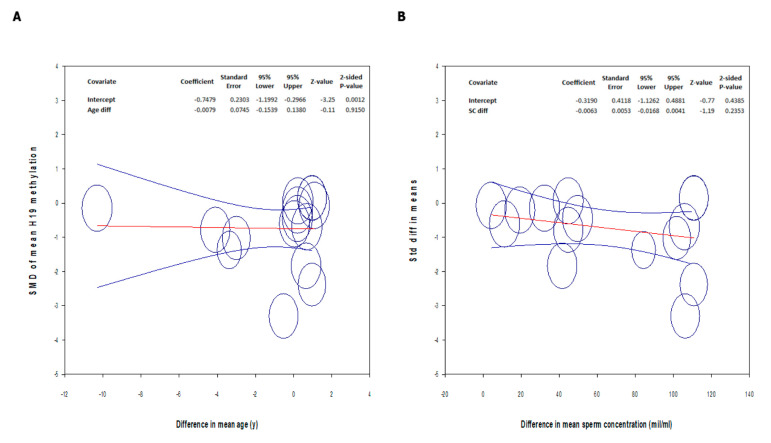
Meta-regression analysis between the standard difference of the mean *H19* gene methylation and the difference in mean age (**A**) and sperm concentration (**B**). Circles represent the studies included in the analysis. The change in the mean and in the 95% CI are represented with the red and the blue lines, respectively.

Meta-regression analysis showed that the difference in the *H19* methylation pattern between the patients and controls was independent of both age and sperm concentration (Figure 4).

## 4. Discussion

In this systematic review and meta-analysis, we demonstrated that patients with infertility and/or abnormal sperm parameters have reduced *H19* gene methylation levels compared to fertile men and/or men with normal sperm parameters. This result confirms findings from the only previous meta-analysis on the topic, which showed lower levels of methylation in infertile patients and patients with oligozoospermia, and an increased risk of DMR methylation aberrations in the *H19* gene in these patients [9].

We performed a subgroup analysis considering the different alterations of sperm parameters and we found that patients with oligozoospermia experienced a significant reduction in *H19* methylation compared to the controls. On the other hand, patients with other alterations in their sperm parameters that did not concern concentration had similar methylation levels, except patients with asthenoteratozoospermia, although only one study analyzed this type of patient. Finally, a statistically significant reduction in *H19* methylation levels was also observed in patients with a history of infertility or RPL compared to fertile men.

The mechanism associating reduced levels of *H19* methylation with oligozoospermia has yet to be fully elucidated. We have recently theorized a role for *IGF2* based on the following considerations. It is known that both the *H19* and *IGF2* genes are located on chromosome 11p15.5 and that their transcription is regulated by H19 DMR [39]. In the maternal allele, the DMR of *H19* is unmethylated, which prevents access to the *IGF2* enhancer. This in turn results in the expression of *H19* and the inhibition of *IGF2*. Conversely, in the paternal allele, *H19* is methylated and this promotes *IGF2* expression and inhibits *H19* expression [40]. In mature spermatogonia, the DMR of *H19* is normally highly methylated, thus favoring *IGF2* expression [41]. Consequently, the reduced DMR methylation of the *H19* gene in patients with oligozoospermia could be associated with a reduced expression of *IGF2* to the degree that spermatogenesis is impaired [42]. In agreement, Sertoli cells produce factors belonging to the IGF family [43] that can promote cell cycle progression by regulating the number of spermatogonia and the final number of spermatozoa [44,45]. In favor of this hypothesis, we have previously reported that spermatozoa express IGF2 messenger RNA and that the levels of this messenger are positively correlated with sperm concentration [42].

The evidence of reduced methylation in the *H19* gene of male partners in couples with RPL is in line with the literature. Indeed, it has been observed that in these men, there is a specific reduction in methylation in some of the CpGs of the DMR of the *H19*/*IGF2* genes compared to fertile men [36]. In this case, an increased SDF rate can be hypothesized. Indeed, a study of 151 normozoospermic patients with unexplained infertility showed that impaired *H19*/*IGF2* methylation, characterized by an increased *H19* expression and a decreased IGF2 expression, correlates with high levels of radical oxygen species; this, in turn, is closely associated with an increased SDF rate [46]. The latter is considered one of the causes of RPL. Accordingly, a meta-analysis of 13 prospective studies showed that the male partners of women with RPL have significantly higher levels of SDF than fertile controls [47].

Finally, in our study, we performed, for the first time, a meta-regression analysis to evaluate the impact of age and sperm concentration on *H19* methylation levels. The results demonstrated that the differences in the paternal age and sperm concentration did not affect the difference in *H19* methylation found between the patients and controls. Several studies in animal models and humans have observed that age is a factor associated with an increase in de novo somatic mutations and alterations of the sperm epigenome. Age affects all known epigenetic mechanisms, including DNA methylation, histone modification, and small non-coding (snc) RNA profiles. It has also been hypothesized that age-induced changes in the sperm epigenome are profound and probably irreversible. However, to date, there has been little research on the effects of age on *H19* gene methylation [48]. A study of 196 adolescent and 176 middle-aged twins failed to find a correlation between age and methylation changes in the DMR of the *H19/IGF2* locus. However, it should be considered that in this study, methylation was evaluated on DNA extracted from whole blood and not from spermatozoa [15]. Our meta-regression results would appear to confirm the absence of the influence of age on DNA methylation results, but further studies are needed to confirm this conclusion. Finally, meta-regression analysis did not detect the influence of sperm concentration and *H19* gene methylation. This would seem to confirm the direct role, not mediated by alterations to the sperm parameters (namely, sperm concentration), of alterations in the methylation of the *H19* gene on the pathogenesis of infertility [23].

Although the conclusion reached by the present study is similar to that of Santi and colleagues, we did observe some important differences between the data of the original studies and those included in the meta-analysis of these colleagues [9]. To address this, we returned directly to the original authors and requested data [22,23,25,26,29]. For other studies, *H19* methylation data were not present in the original articles and, in receiving responses from the authors of these original articles [27,30], we noted that the data differed from those reported in the previous study [9]. Furthermore, some authors replied that in the original study, they did not evaluate the global levels of methylation, but that they calculated the percentage of patients who did not have complete *H19* methylation [31,32,49]. Another study lacked a control group [33]. For this reason, we did not include these studies in our meta-analysis. Finally, since azoospermia was an exclusion criterion, we did not include the study by Minor and colleagues [20]. For age and sperm concentration data, we also observed similar differences [21,28,29,34]. Supplementary Table S3 summarizes all the differences between the original data and those reported in the previous meta-analysis [9]. Moreover, Supplementary Table S4 reports the responses provided by the authors of the original authors to our queries.

Another interesting aspect that could be considered in future studies is the role that abnormal sperm epigenetics may play in the controversial association between ART and the adverse outcome of offspring conceived using these techniques [8]. According to a recent systematic review and meta-analysis, ART may affect epigenetics, including DNA methylation, in the fetus and the placenta. This could be associated with the manipulation and processes used in these techniques [50]. However, based on the results of the present study, the possibility that the association between ART and aberrant DNA methylation at imprinted loci may be due to the already altered methylation pattern of paternal gametes should be considered. The *H19* methylation status could be preliminarily assessed in patients seeking ART to predict its outcomes in terms of the birth rate and offspring health. Indeed, several imprinting disorders, such as the Prader-Willi, Angelmann, Beckwith-Wiedemann, and Silver-Russell syndromes, appear to be related to ART use [51]. In particular, evidence in the literature suggests that *H19* is hypomethylated more in ART offspring compared to spontaneously conceived offspring [8]. Likewise, the epigenetic alteration in infertile male gametes consists of *H19* hypomethylation. In the future, prospective studies will be needed to implement a cost-effective panel of genes to be assessed in the sperm of patients undergoing ART, in order to predict the success rate of the technique, as well as the health of the offspring. Based on the results of the present analysis, the *H19* gene could be included in such genetic panels. The presence of hypomethylation in the sperm might indeed suggest a lower ART success, and a greater risk of methylation aberrations in the offspring.

To our knowledge, this study represents the second meta-analysis performed so far in order to investigate *H19* methylation levels in the spermatozoa of infertile patients. This meta-analysis has some limitations. First, this is a meta-analysis of cross-sectional studies, which is why a direct cause-and-effect relationship between *H19* gene methylation levels and infertility cannot be established. Second, the statistical analysis showed significant heterogeneity across studies. This heterogeneity can likely be attributed to differences between the populations examined, although subgroup analyses allowed us to study methylation levels in different infertility subgroups. Finally, another limitation of this meta-analysis is the difference in the methodologies applied in the studies included to measure the DNA methylation levels. Moreover, in some cases, there was a difference in the number of specific CpGs in the DMRs of the *H19* gene that was analyzed. Therefore, to soften this limitation, the results are expressed as SMD.

## 5. Conclusions

The results of the present study show that patients experiencing infertility and with oligozoospermia have lower *H19* gene methylation levels than those without. These results remain significant after correcting for age and sperm concentration between the two groups, thus confirming a direct correlation with the different *H19* gene methylation patterns. Within the limitations of the published data of variable quality, our meta-analysis suggests the potential causal role of reduced levels of *H19* gene methylation in the pathogenesis of infertility, particularly in patients with oligozoospermia. Interestingly, *H19* hypomethylation has been reported in ART-conceived offspring [8]. Whether this depends on ART manipulation or on the greater rate of aberrations in the methylation of the *H19* gene in the sperm of patients accessing ART needs to be elucidated. Based on the results of the present study, the *H19* gene could be included in the genetic panel of prospective studies aimed at identifying the most representative and cost-effective genes to be analyzed in couples undergoing ART.

## Figures and Tables

**Table 1:** Inclusion and exclusion criteria according to the PECOS model [18].

	Inclusion Criteria	Exclusion Criteria
**Population**	Male patients	Adolescents, women, and patients with azoospermia
**Exposure**	Abnormal sperm parameters (oligo and/or astheno- and/or terato-zoospermia), infertile men, or recurrent pregnancy loss	
**Comparison**	Normal sperm parameters (normozoospermia) or fertile men	
**Outcomes**	Methylation of *H19* differentially-methylated region	
**Study type**	Observational studies, randomized controlled studies, case-control studies	Animal studies, in vitro studies, review and meta-analyses, case reports, book chapters, editorials

**Table 2:** Main characteristics of included cross-sectional studies.

Author	Cases Sample Size	Control Sample Size	Mean Age of Cases	Mean Age of Controls	Mean Sperm Concentration Cases	Mean Sperm Concentration Controls	Outcome Assessed	Method of Evaluation of the Outcome
Boissonnas et al., 2010 [34]	22 Oligo-astheno-teratozoospermic patients	17 Normozoospermic patients	NR	NR	NR	118.9 ± 28.7	Methylation of CTCF-3	Bisulfite modification, PCR and pyrosequencing
							Methylation of CTCF-6	
	19 Teratozoospermiac patients	17 Normozoospermic patients	NR	NR	164.6 ± 50	118.9 ± 28.7	Methylation of CTCF-3	Bisulfite modification, PCR and pyrosequencing
							Methylation of CTCF-6	
El-Hajj et al., 2011 [28]	106 infertile patients	28 fertile patients	38.1 ± 5.62	38.33 ± 5.59	11.41 ± 5.88	56.22 ± 24.14	Methylation of the 4 CpG of *H19* ICR	Bisulfite Pyrosequencing
Ankolkar et al., 2012 [26]	26 patients with history of recurrent pregnancy loss	26 fertile patients	35.4 ± 4.53	31.3 ± 4.49	64.5 ± 25.49	76.1 ± 64.16	Methylation of the whole *H19* ICR	Bisulfite modification and PCR
							Percentage of clones fully methylated	
							Methylation of CTCF-6	
Camprubí et al., 2012 [27]	107 infertile patients	30 fertile patients	36.4 ± 5.60	26 ± 6.15	54.1 ± 46.8	86.42 ± 36.88	Methylation of the whole *H19* ICR	Bisulfite pyrosequencing
Li et al., 2013 [22]	20 Oligozoospermic patients	20 Normozoospermic patients	31.25 ± 5.63	31.85 ± 3.88	5.22 ± 3.33	101.99 ± 35.63	Methylation of the whole *H19* ICR	Bisulfite modification and PCR
							Methylation of CTCF-6	
	20 Asthenozoospermic patients	20 Normozoospermic patients	32.95 ± 5.21	31.85 ± 3.88	84.19 ± 33.12	101.99 ± 35.63	Methylation of the whole *H19* ICR	
							Methylation of CTCF-6	
Laurentino et al., 2015 [29]	7 Oligo-astheno-teratozoospermic patients	5 Normozoospermic patients	35.57 ± 5.65	32.2 ± 2.59	0.79 ± 0.78	85.18 ± 66.61	Methylation of CTCF-6	Bisulfite Pyrosequencing
Montjean et al., 2015 [30]	30 Oligozoospermic patients	62 Normozoospermic controls	38.3 ± 6	38.5 ± 5.3	5.8 ± 3.9	55.7 ± 43	Methylation of the whole *H19* ICR	Bisulfite modification and PCR
Xu et al., 2016 [25]	46 Asthenozoospermic patients	49 Normozoospermic patients	31.95 ± 21.77	32.16 ± 22.82	43.93 ± 22.86	63.31 ± 22.89	Methylation of 14 CpG of the *H19* ICR	Bisulfite conversion and MassARRAY quantitative methylation analysis
Li et al., 2016 [23]	15 infertile patients	15 fertile patients	35.5 ± 8.5	32.5 ± 6.5	11.8 ± 7.2	113.6 ± 32.1	Methylation of the whole *H19* ICR	PCR
Dong et al., 2017 [21]	48 Oligozoospermic patients	50 Normozoospermic patients	31.52 ± 3.58	32.22 ± 3.59	10.9 ± 3.86	115.98 ± 31.12	Methylation of the whole *H19* ICR	Bisulfite modification, PCR and pyrosequencing
	52 Asthenozoospermic patients		32.17 ± 3.27	32.22 ± 3.59	104.62 ± 29.49			
	55 Teratozoospermic patients		31.13 ± 3.34	32.22 ± 3.59	111.63 ± 30			
Peng et al., 2018 [24]	39 Oligoasthenozoospermic patients	50 Normozoospermic patients	32.74 ± 5.85	32.22 ± 3.59	9.72 ± 5.72	115.98 ± 31.12	Methylation of 16 CpG of the *H19* ICR	Bisulfite pyrosequencing
	36 Asthenoteratozoospermic patients	50 Normozoospermic patients	31.56 ± 5.78	32.22 ± 3.59	74.35 ± 65.39	115.98 ± 31.12		

**Table 3:** Quality of Evidence of the included studies according to the Cambridge Quality Checklists.

Authors	Checklist for Correlates	Checklist for Risk Factor	Checklist for Causal Risk Factors	Total
Ankolkar et al., 2012 [26]	2	1	2	5
Boissonnas et al., 2010 [34]	2	1	2	5
Camprubí et al., 2012 [27]	2	1	2	5
Dong et al., 2017 [21]	2	1	2	5
El-Hajj et al., 2011 [28]	2	1	2	5
Laurentino et al., 2015 [29]	1	1	2	4
Li et al., 2013 [22]	2	1	2	5
Li et al., 2016 [23]	2	1	2	5
Montjean et al., 2015 [30]	2	1	1	4
Peng et al., 2018 [24]	2	1	2	5
Xu et al., 2016 [25]	2	1	2	5

## Data Availability

Data will be made available upon request to the corresponding author.

## Supplementary Materials

The following supporting information can be downloaded at: https://www.mdpi.com/article/10.3390/ijms24087224/s1.
