# Supplementary material for: H19 Sperm Methylation in Male Infertility: A Systematic Review and Meta-Analysis
Source: Int J Mol Sci. 2023 Apr 13;24(8):7224. doi: 10.3390/ijms24087224 (PMC10139270; doi:10.3390/ijms24087224)
Supplement: Supplementary file 1 [file ijms-24-07224-s001.zip › ijms-2320467-supplementary.pdf]

International Journal of Molecular Sciences

MDPI

Supplementary Table S1. MOOSE Checklist for Meta-analyses of Observational Studies

Item No	Recommendation	Reported on Page No
Reporting of background should include		
1	Problem definition	4
2	Hypothesis statement	5
3	Description of study outcome(s)	6
4	Type of exposure or intervention used	6
5	Type of study designs used	6
6	Study population	6
Reporting of search strategy should include		
7	Qualifications of searchers (eg, librarians and investigators)	6
8	Search strategy, including time period included in the synthesis and key words	6
9	Effort to include all available studies, including contact with authors	6
10	Databases and registries searched	6
11	Search software used, name and version, including special features used (eg, explosion)	6
12	Use of hand searching (eg, reference lists of obtained articles)	6
13	List of citations located and those excluded, including justification	6
14	Method of addressing articles published in languages other than English	-
15	Method of handling abstracts and unpublished studies	-
16	Description of any contact with authors	6
Reporting of methods should include		
17	Description of relevance or appropriateness of studies assembled for assessing the hypothesis to be tested	6
18	Rationale for the selection and coding of data (eg, sound clinical principles or convenience)	6
19	Documentation of how data were classified and coded (eg, multiple raters, blinding and interrater reliability)	6
20	Assessment of confounding (eg, comparability of cases and controls in studies where appropriate)	6
21	Assessment of study quality, including blinding of quality assessors, stratification or regression on possible predictors of study results	7
22	Assessment of heterogeneity	7
23	Description of statistical methods (eg, complete description of fixed or random effects models, justification of whether the chosen models account for predictors of study results, dose-response models, or cumulative meta-analysis) in sufficient detail to be replicated	7
24	Provision of appropriate tables and graphics	Table 1-2

		Supp. Tab S1-S4 Figs 1-4
Reporting of results should include		
25	Graphic summarizing individual study estimates and overall estimate	Figs 2
26	Table giving descriptive information for each study included	Table 2
27	Results of sensitivity testing (eg, subgroup analysis)	9-10, Figs 2-3
28	Indication of statistical uncertainty of findings	8-10, Figs 2-4

**Item No**	**Recommendation**	**Reported on Page No**
Reporting of discussion should include		
29	Quantitative assessment of bias (eg, publication bias)	10, Figure 3
30	Justification for exclusion (eg, exclusion of non-English language citations)	8
31	Assessment of quality of included studies	9, Table 3
Reporting of conclusions should include		
32	Consideration of alternative explanations for observed results	11-13
33	Generalization of the conclusions (ie, appropriate for the data presented and within the domain of the literature review)	11-13
34	Guidelines for future research	11-13
35	Disclosure of funding source	-

From: Stroup DF, Berlin JA, Morton SC, et al, for the Meta-analysis Of Observational Studies in Epidemiology (MOOSE) Group. Meta-analysis of Observational Studies in Epidemiology. A Proposal for Reporting. *JAMA*. 2000;283(15):2008-2012. doi: 10.1001/jama.283.15.2008.

International Journal of *Molecular Sciences*

MDPI

Supplementary Table S2. PRISMA 2009 Checklist

Section/topic	#	Checklist item	Reported on page #
**TITLE**			
Title	1	Identify the report as a systematic review, meta-analysis, or both.	1
**ABSTRACT**			
Structured summary	2	Provide a structured summary including, as applicable: background; objectives; data sources; study eligibility criteria, participants, and interventions; study appraisal and synthesis methods; results; limitations; conclusions and implications of key findings; systematic review registration number.	3
**INTRODUCTION**			
Rationale	3	Describe the rationale for the review in the context of what is already known.	4-5
Objectives	4	Provide an explicit statement of questions being addressed with reference to participants, interventions, comparisons, outcomes, and study design (PICOS).	5
**METHODS**			
Protocol and registration	5	Indicate if a review protocol exists, if and where it can be accessed (e.g., Web address), and, if available, provide registration information including registration number.	/
Eligibility criteria	6	Specify study characteristics (e.g., PICOS, length of follow-up) and report characteristics (e.g., years considered, language, publication status) used as criteria for eligibility, giving rationale.	6
Information sources	7	Describe all information sources (e.g., databases with dates of coverage, contact with study authors to identify additional studies) in the search and date last searched.	6
Search	8	Present full electronic search strategy for at least one database, including any limits used, such that it could be repeated.	6

Study selection	9	State the process for selecting studies (i.e., screening, eligibility, included in systematic review, and, if applicable, included in the meta-analysis).	6
Data collection process	10	Describe method of data extraction from reports (e.g., piloted forms, independently, in duplicate) and any processes for obtaining and confirming data from investigators.	6
Data items	11	List and define all variables for which data were sought (e.g., PICOS, funding sources) and any assumptions and simplifications made.	6
Risk of bias in individual studies	12	Describe methods used for assessing risk of bias of individual studies (including specification of whether this was done at the study or outcome level), and how this information is to be used in any data synthesis.	7
Summary measures	13	State the principal summary measures (e.g., risk ratio, difference in means).	7
Synthesis of results	14	Describe the methods of handling data and combining results of studies, if done, including measures of consistency (e.g., I^2) for each meta-analysis.	7

Supplementary Table S3. Discrepancies found in the meta-analysis by Santi and colleagues

Author	Discrepancies found
Ankolkar et al., 2012	• Median value of the original article considered and used as the mean
Boissonnas et al., 2010	• Difference in the sperm concentration value of normozoospermic controls reported by Santi and colleagues compared to the value reported in the original article
Camprubí et al., 2012	• Diversity of *H19* gene methylation values, age, and sperm concentration of cases and controls reported by Santi and colleagues compared to those sent to us by the authors of the original article (see Supplementary Table S4)
Dong et al., 2016	• Difference in age and sperm concentration of normozoospermic patients reported by Santi and colleagues compared to the found in the original article
El-Hajj et al., 2011	• Difference in age and sperm concentration of the cases and controls reported by Santi and colleagues compared to those sent to us by the authors of the original article (see Supplementary Table S4)
Laurentino et al., 2015	• Difference in age and sperm concentration of cases and controls reported by Santi and colleagues compared to those sent to us by the authors of the original article (see Supplementary Table S4)<br>• Difference in standard deviation of methylation gene of *H19* reported by Santi and colleagues compared to that reported in the original article
Li et al., 2013	• Difference in *H19* gene methylation value reported by Santi and colleagues compared to those reported in the original article
Li et al., 2016	• Difference in H19 gene methylation value reported by Santi and colleagues compared to those reported in the original article
Marques et al., 2004	• The authors did not evaluate the methylation values of *H19* but the percentage of patients with methylated clones (see Supplementary Table S4). Therefore, the data cannot be included
Marques et al., 2008	• The authors did not evaluate the methylation values of *H19* but the percentage of patients with methylated clones (see Supplementary Table S4). Therefore, the data cannot be included
Montjean et al., 2015	• Difference in *H19* gene methylation value compared to those sent by the authors of the original article (see Supplementary Table S4)<br>• Difference in age and sperm concentration of controls compared to the original article
Tian et al., 2014	• As reported in the original article and also confirmed by the authors we contacted by email (see Supplementary Table S4), it is a cohort study
Xu et al., 2016	• The standard error of the mean was used as the standard deviation

Supplementary Table S4. Answers from the corresponding authors of the original article.

Article	Request	Answer
Ankolkar et al., 2012	SD of the age of cases and controls	Dear Dr. Cannarella<br>Please find the required information on Std Dev for age of male partner<br>Male age<br>CONTROL RSA<br>N 26 26<br>Mean 31.3 35.44<br>SD 4.489 4.535<br>I hope this information will be useful to you<br>Thanking you. Best regards. Nafisa Balasinor
Camprubí et al., 2012	Methylation levels of H19 in the sperm (mean, SD or median, IQR) of patient and control groups.<br>Age and sperm concentration (mean, SD or median, IQR) of patient and control groups	Dear Rossella, thank you for considering our article eligible for your systematic review.<br>I looked through the files associated with this project and I have extracted the information you requested in three documents (enclosed):<br>1. Controls' and patients' ages and seminal parameters<br>2. Methylation levels of each CpG from both loci in controls<br>3. Methylation levels of each CpG from both loci in infertile patients.<br>I think it will be easy for you to obtain the values you need from the data gathered in the tables. In any case, if you have any trouble, please do not hesitate to contact me again.<br>Sincerely yours. Joan Blanco Rodríguez<br>Professor Agregat
El-Hajj et al., 2011	Age and sperm concentration (mean, SD or median, IQR) of the patient and control group	Dear Rosella, I hope you are doing well. Please find the requested information below. Regarding age, the birthdate was not available for 5 samples from the normozoospermia group, however it was available for all the oligozoospermic samples. Hope this helps. Best,<br>Nady.<br>Age:<br>Average Normozoospermia: 38.3287671<br>Stdev: 5.59030608

		Q1:34<br>Q3: 42<br>IQR:8<br>Average Oligozoospermia: 38.1111111<br>Stdev: 5.62572178<br>Q1:34<br>Q3:41.5<br>IQR:6.5<br>Sperm Count (mil/ml)<br>Average Normozoospermia: 56.21794872<br>Stdev: 24.14063096<br>Q1: 40<br>Q3: 70<br>IQR: 30<br>Average Oligozoospermia: 11.41269841<br>Stdev: 5.876785715<br>Q1: 7<br>Q3: 17<br>IQR: 10
Laurentino et al., 2015	We asked to provide age and sperm concentration of the 5 normozoospermic and 7 OAT patients included in Table 1	Dear Rossella, I'm sorry it took so long, I was on holidays. You can find a table attached with the age and sperm of each of the patients in the two groups. Please note that one of the normozoospermic patients (N1) had low concentration. We still took him as part of the normal group as the total sperm count was perfectly normal, as well as all other parameters. Please let me know if you need any further information and good luck with the data analysis. Best, Sandra
Marques et al., 2004	Methylation levels of H19 in the sperm (mean, SD or median, IQR) of patient and control groups	Dear Prof. Cannarella, We were able to recollect almost all the data regarding age of the patients at the time of sperm collection but some values are missing, please see the table attached (MV - Missing Values). Regarding the methylation values, I am not sure how to address that question because of the following - in this first paper (Lancet 2004), we analysed methylation by direct sequencing (following bisulfite modification); hence, we calculated the percentage of patients showing complete methylation of H19

	Age and sperm concentration (mean, SD or median, IQR) of patient and control groups	(whether the 18 CpGs methylated or just CpG7 /unmethylated - Fig. A - since this was shown to constitute a SNP between C and T and we cannot ascertain its methylation status). For the normozoospermic group, all the individuals showed complete methylation of H19; in the oligozoospermic group, 23 out of 96 patients showed incomplete methylation (corresponding to 1 to 5 CpGs unmethylated or having a double peak of methylation and unmethylation (double peak of cytosine and thymine at the same CpG, in the direct sequencing). Hence, in this group, 24% (16-34%, 95% Confidence interval) showed incomplete methylation, with 17% (8/46) in the moderate oligozoospermia group and 30% (15/50) in the severe oligozoospermia group.<br>Because we performed direct sequencing, it is not straightforward to calculate methylation values in the sperm as you requested but I calculated H19 methylation levels for each patient based on the number of unmethylated or double peak (unmethylated and methylated) cytosines as you can find in the table attached (highlighted in blue and yellow) but for the above-mentioned reasons I am not sure this is the correct approach. We have another paper, in Mol Hum Reprod 2008, where we have a more detailed analysis by cloning and sequencing (please find it attached). Let me know if I can be of any further help. Best regards, Joana Marques
Marques et al., 2008	Methylation levels of H19 in the sperm (mean, SD or median, IQR) of patient and control groups<br>Age and sperm concentration (mean, SD or median, IQR) of patient and control groups	Dear Prof. Cannarella, We were able to recollect almost all the data regarding age of the patients at the time of sperm collection but some values are missing, please see the table attached (MV - Missing Values). Regarding the methylation values, I am not sure how to address that question because of the following - in this first paper (Lancet 2004), we analysed methylation by direct sequencing (following bisulfite modification); hence, we calculated the percentage of patients showing complete methylation of H19 (whether the 18 CpGs methylated or just CpG7 /unmethylated - Fig. A - since this was shown to constitute a SNP between C and T and we cannot ascertain its methylation status). For the normozoospermic group, all the individuals showed complete methylation of H19; in the oligozoospermic group, 23 out of 96 patients showed incomplete methylation (corresponding to 1 to 5 CpGs unmethylated or having a double peak of methylation and unmethylation (double peak of cytosine and thymine at the same CpG, in the direct sequencing). Hence, in this group, 24% (16-34%, 95% Confidence interval) showed incomplete methylation, with 17% (8/46) in the moderate oligozoospermia group and 30% (15/50) in the severe oligozoospermia group.

		Because we performed direct sequencing, it is not straightforward to calculate methylation values in the sperm as you requested but I calculated H19 methylation levels for each patient based on the number of unmethylated or double peak (unmethylated and methylated) cytosines as you can find in the table attached (highlighted in blue and yellow) but for the above-mentioned reasons I am not sure this is the correct approach. We have another paper, in Mol Hum Reprod 2008, where we have a more detailed analysis by cloning and sequencing (please find it attached). Let me know if I can be of any further help. Best regards, Joana Marques
Montjean et al., 2013	Methylation levels of H19 in the sperm (mean, SD or median, IQR) of fertile and infertile patients<br>Age and sperm concentration (mean, SD or median, IQR) of the infertile group	Dear Rossella. In this study. The mean age was 39.3+/-4.7 in Normospermic vs 37.6+/-6.1 in Oligozoospermic patients The mean sperm concentration was 63.7+/-48.8 in normospermic vs 8.4+/-6.3 in oligospermic patients The global methylation level was not assessed. The methylation errors in H19 and MEST DMR are described in the tables. I hope this helps Best regards. Debbie Montjean, PhD
Montjean et al., 2015	Methylation levels of H19 in the sperm (mean, SD or median, IQR) of fertile and infertile patients.<br>Age and sperm concentration (mean, SD or median, IQR) of the infertile group	Dear Rossella. Thanks for including this article in your Meta-Analysis. The 2 groups compared in this article were Normopermic and Oligospermic men. The methylation levels of H19 in the sperm of the normospermic was (mean, SD) 26.6,24.9 and 16.4, 15.8 in Oligospermic patients. The mean age was 38.5+/-5.3 in Normospermic vs 38.3+/-6.0 in Oligozoospermic patients. The mean sperm concentration was 55.7+/-43.0 in normospermic vs 5.8+/-3.9 in oligospermic patients. I hope this will help you. Best regards. Debbie Montjean
Tian et al., 2014	Methylation levels of H19<br>in the sperm of fertile patients (mean, SD or median, IQR)<br>Sperm concentration of fertile patients	Dear Cannarella, Thanks for your email. I am Dr. Meiping Tian (first author). The male participants recruited from reproduction department who were undergoing fertility assessment because of an idiopathic inability to conceive. So the participants were clinical population. Unfortunately, we are unable to discriminate between infertile and fertile because of without further clinical follow-up. We have just listed all the participants semen parameters and sperm DNA methylation in the Table 3. Best regards, Meiping Tian
